# Gene Repression in Haloarchaea Using the CRISPR (Clustered Regularly Interspaced Short Palindromic Repeats)-Cas I-B System*

**DOI:** 10.1074/jbc.M116.724062

**Published:** 2016-05-16

**Authors:** Aris-Edda Stachler, Anita Marchfelder

**Affiliations:** From the Department of Biology II, Ulm University, 89069 Ulm, Germany

**Keywords:** archaea, CRISPR/Cas, crRNA, gene silencing, Haloferax volcanii, CRISPRi, type I-B

## Abstract

The clustered regularly interspaced short palindromic repeats (CRISPR)-Cas system is used by bacteria and archaea to fend off foreign genetic elements. Since its discovery it has been developed into numerous applications like genome editing and regulation of transcription in eukaryotes and bacteria. For archaea currently no tools for transcriptional repression exist. Because molecular biology analyses in archaea become more and more widespread such a tool is vital for investigating the biological function of essential genes in archaea. Here we use the model archaeon *Haloferax volcanii* to demonstrate that its endogenous CRISPR-Cas system I-B can be harnessed to repress gene expression in archaea. Deletion of *cas3* and *cas6b* genes results in efficient repression of transcription. crRNAs targeting the promoter region reduced transcript levels down to 8%. crRNAs targeting the reading frame have only slight impact on transcription. crRNAs that target the coding strand repress expression only down to 88%, whereas crRNAs targeting the template strand repress expression down to 8%. Repression of an essential gene results in reduction of transcription levels down to 22%. Targeting efficiencies can be enhanced by expressing a catalytically inactive Cas3 mutant. Genes can be targeted on plasmids or on the chromosome, they can be monocistronic or part of a polycistronic operon.

## Introduction

**FIGURE 1.**
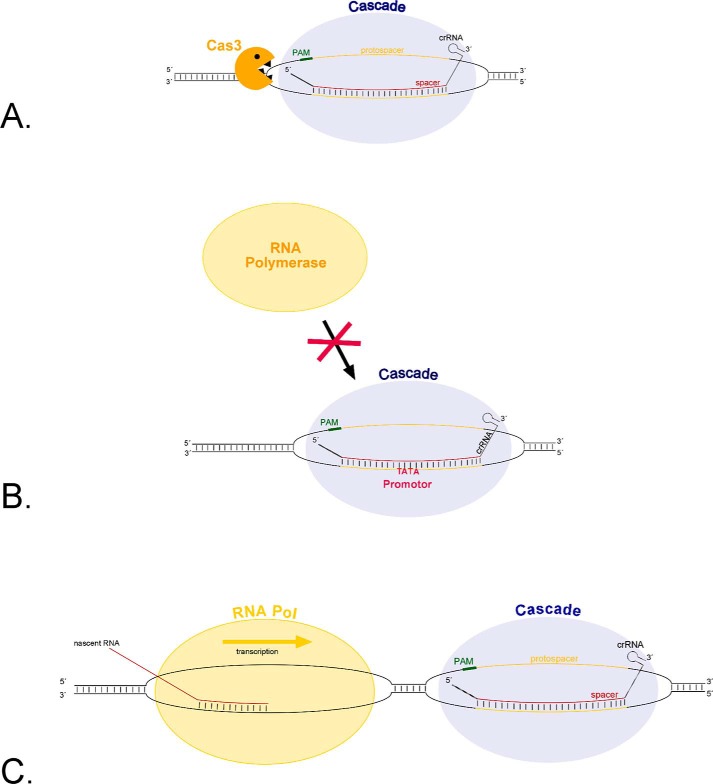
**Schematic drawing of the CRISPR-Cas type I-B CRISPRi tool.** *A,* interference reaction of CRISPR-Cas type I. Cascade is guided by the crRNA to the target DNA sequence. After binding of Cascade, Cas3 is recruited to cleave the target DNA. *B,* inhibition of transcription initiation by CRISPRi. By using a crRNA that binds to the promoter region, Cascade binds together with the crRNA to the promoter region, if Cas3 is missing, the DNA is not cleaved but binding of transcription factors and RNA polymerase is blocked, thereby preventing transcription initiation. *C,* inhibition of transcription elongation by CRISPRi. If Cascade is directed by the crRNA to a target sequence within the open reading frame of a gene, it can act as roadblock for the RNA polymerase and stop transcription elongation.

CRISPR-Cas^2^ (Clustered Regularly Interspaced Short Palindromic Repeats)-CRISPR associated) is a defense mechanism found in bacteria and archaea to fend off foreign genetic elements (for recent reviews, see Refs. 1–5). Many different versions of the CRISPR-Cas system have been discovered and they have been grouped into two classes and six major types (type I-VI) that are subdivided into several subtypes (I-A-F, I-U, II-A-C, III-A-D, and V-A-B) (6, 7). All CRISPR-Cas types have two components in common that are essential for the function of this defense mechanism: the Cas proteins and the crRNA (CRISPR RNA). The Cas proteins are guided by the crRNA to a specific target sequence that is subsequently cleaved by the Cas proteins. Since its discovery the CRISPR-Cas system has been developed into different molecular biology tools, *e.g.* for genome editing or transcription regulation in bacteria and eukarya (8–11). Especially the type II CRISPR-Cas9 system has become a vast resource for numerous applications (12, 13). One of these applications is termed CRISPR interference (CRISPRi) after the eukaryotic RNAi approach. It employs the CRISPR-Cas type II system (also called CRISPR-Cas9) for down-regulating gene expression (14). For this application a catalytically inactive version of the *Streptococcus pyogenes* Cas9 protein (dCas9) was generated that binds to the target DNA but is not able to cleave the DNA (14). The CRISPR-Cas9 CRISPRi tool has been shown to efficiently down-regulate genes in eukaryotes and it was also used efficiently in bacteria (14, 15). Another CRISPR-Cas system (type III-B) has been developed to target and degrade mRNAs and thus is applicable for targeted mRNA degradation (16–18). For this application the Cas protein complex from type III is required, thus it is most convenient to use in cells having an endogenous CRISPR-Cas type III system. In *Escherichia coli* a CRISPR-Cas type I system was developed into a CRISPRi tool (19, 20). Here, the endogenous CRISPR-Cas type I-E system was harnessed and modified to use for transcription regulation. In the CRISPR-Cas type I-E system the Cas protein complex (called Cascade for Cas protein complex for antiviral defence) binds via the crRNA to the target DNA and recruits the Cas3 protein to degrade the target DNA (5, 21, 22). To prevent target DNA degradation a *cas3* gene deletion strain was generated, resulting in a strain that had a functional Cascade complex binding to specific DNA sequences but unable to cleave those sequences (19, 20) (Fig. 1).

**FIGURE 2.**
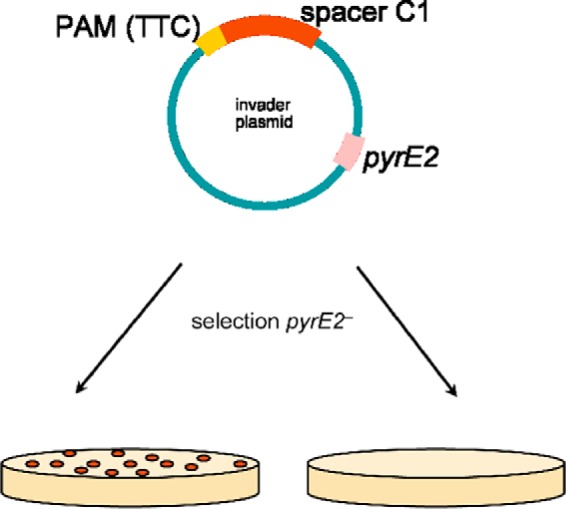
**The plasmid-based interference test.** The spacer sequence C1 (spacer1 from *Haloferax* CRISPR locus C) was cloned adjacent to one of the PAM sequences identified for *Haloferax* (TTC) into the vector pTA409 yielding the plasmid invader pTA409-PAM3-C1. The *Haloferax* strain was transformed with a crRNA expressing plasmid and subsequently with the plasmid invader (pTA409-PAM3-C1). Selection for transformants was achieved by growth without uracil, which is only possible when cells contain the *pyrE2* selection marker encoded on the vector. Cells can grow if the interference is not active and therefore the invader plasmid is retained. If the plasmid is recognized by the CRISPR-Cas system and degraded, cells cannot grow on selective media due to loss of the selection marker.

For archaea a tool to down-regulate transcription is currently not available. Because more and more archaeal organisms are studied (23), a method to down-regulate genes in archaea is an important tool to further analyze gene functions in this domain. The prevalent subtypes of the CRISPR-Cas I type found in archaea are subtypes I-A, I-B, and I-D (24, 25). Therefore, we developed the type I-B system into a CRISPRi tool for down-regulating archaeal genes. Because many archaea have the type I-B system it is possible to harness the endogenous system for the CRISPRi approach and to convert it into a transcriptional regulator. Here, we used the type I-B system of the model archaeon *Haloferax volcanii*, the best characterized type I-B system in a genetically tractable archaeon, to generate the I-B CRISPRi tool. The *Haloferax* type I-B has already been characterized in detail (26–29). It consists of eight Cas proteins (Cas1–5, Cas6b, Cas7, and Cas8b) and three CRISPR loci that are constitutively expressed (30). The interaction between the crRNA and the target DNA requires a 10-nucleotide long seed sequence (31). The Cascade core complex consists of Cas5 and Cas7, Cas8b is only loosely associated (32, 33), and Cas6b is dispensable for the interference reaction (34). A prerequisite for binding of Cascade to a target sequence is the presence of a short motif in the invader sequence: the protospacer adjacent motif (PAM), it is located directly adjacent to the sequence targeted in the DNA (Figs. 1 and 2). The *Haloferax* defense system is able to detect invaders with six different PAM (30). The broad PAM recognition potential of the I-B system is a great advantage for establishing this system as CRISPRi tool. Most systems analyzed to date recognize fewer PAM sequences. The fact that the *Haloferax* type I-B system recognizes six PAM sequences makes the selection of target sequences in the gene to be down-regulated much easier. Furthermore, for *Haloferax* a Cas6b independent way of crRNA production was developed making crRNA production easier and allowing interference reactions in a Δ*cas6b* strain (34).

Here, we show that transcriptional down-regulation with a type I-B system works efficiently. Deletion of the genes for the Cas3 and Cas6b proteins and expression of specific crRNAs result in repression of the reporter gene. Genes can be targeted on a plasmid or on the chromosome, they can be monocistronic or part of a polycistronic operon, essential genes can also be repressed. To facilitate easier design of crRNAs we generated different crRNA mutants to define the shortest functional crRNA. Repression efficiencies can be enhanced by addition of a Cas3 mutant.

## Results and Discussion

### 

#### 

##### Prerequisites for a CRISPRi System

*H. volcanii* encodes a CRISPR-Cas type I-B system, which uses the Cas3 protein to initiate degradation of the target DNA. To use the endogenous system for gene repression we deleted the *cas3* gene, using the pop-in/pop-out method to remove the complete open reading frame of the *cas3* gene (35, 36) generating *Haloferax* strain HV28 (Δ*cas3*). To verify that this deletion strain can no longer process the target DNA we employed an interference test (30) (Fig. 2). In this test a plasmid is used as an invader that can be targeted by an endogenous crRNA. If the plasmid invader is recognized by the CRISPR-Cas system it is degraded and a drastic reduction in the number of transformants is observed (30). The Δ*cas3* strain should not be able to degrade the invader plasmid, because it is lacking the endonuclease degrading the target DNA (Cas3). Transformation of wild type *Haloferax* cells and Δ*cas3* cells clearly shows that the deletion mutant cannot fend off the invader (Table 1). To monitor the effects of down-regulation of gene expression we used the *Haloferax alicantei* β-galactosidase reporter gene (*bgaHa*) encoded on plasmid pTA599 as target (37). The *H. alicantei* β-galactosidase shows higher activity in protein assays than the endogenous *H. volcanii* β-galactosidase. To prevent homologous recombination between the *H. alicantei* β-galactosidase gene with the similar *H. volcanii* β-galactosidase gene the *H. volcanii* β-galactosidase gene was deleted in strain HV28, generating strain HV29 (Δ*cas3*Δ*bgaH*).

**TABLE 1:** **The Cas3 protein is essential for the interference reaction** A plasmid invader assay was used to determine the interference activity of different *Haloferax* strains. Strains were transformed with the invader plasmid pTA409-PAM3-C1 (31). Cells that have an active interference system degrade the plasmid and cannot grow on selective medium, resulting in drastically reduced transformation rates (reduced by at least factor 0.01) (30). The wild type strain is active in interference, transformation rates of the invader plasmid are reduced by factor 0.001. The strain without Cas3 (HV28) is not active in interference, transformation rates of the invader plasmid are not reduced. If strain HV28 is complemented with the *cas3* gene on a plasmid (HV28 × pTA927-Cas3-Flag-N), the interference activity is restored again, showing that Cas3 is essential for an active interference.

Strain	Reduction in transformation rate by factor
Wild type (H119)	0.001*^a^*
HV28 (Δ*cas3*)	1.3 (no reduction, no interference)
HV28 × pTA927-Cas3-Flag-N	0.002

*^a^* Data taken from Maier *et al.* (31).

##### A CRISPR-Cas Type I-B Can Repress Expression of a Reporter Gene

**FIGURE 3.**
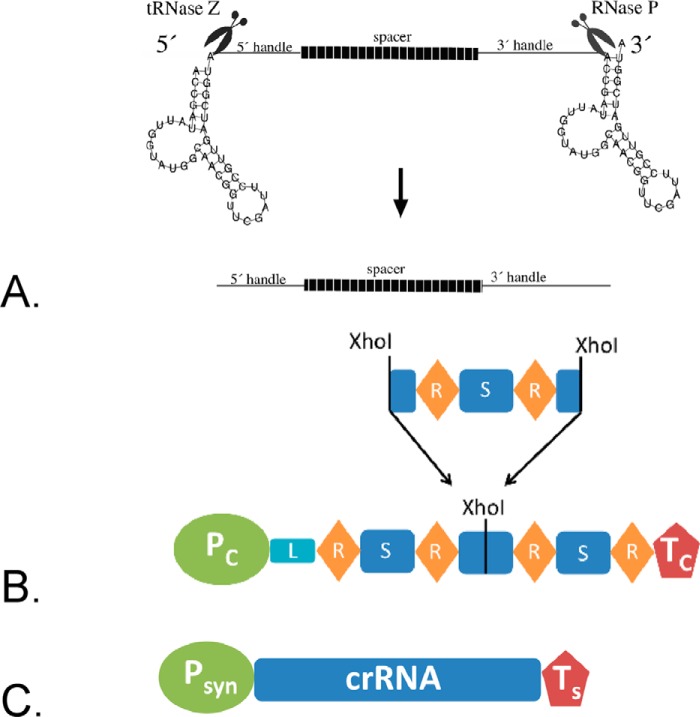
**Generation of crRNAs.** *A,* tRNase Z and RNase P release the crRNA from the precursor in the tRNA-like-element construct. The transcript expressed from the plasmid contains two tRNA-like elements flanking the crRNA gene. The tRNA-like elements are processed by tRNase Z and RNase P resulting in a crRNA with the natural 5′ and 3′ handles (34). *B,* crRNA expression from the S-plasmid. The S-plasmid contains part of the CRISPR locus C from *Haloferax*: the promoter (*P_C_*), the leader sequence (*L*), the terminator (*T*), and four repeats (*R*) as well as three spacer (*S*) sequences. The second spacer has been mutated to contain a XhoI cleavage site. New spacers can be inserted as shown. *C,* expression using a synthetic promoter and terminator. The crRNA is expressed from the synthetic promoter p.syn and terminated by the synthetic terminator (46).

**FIGURE 4.**
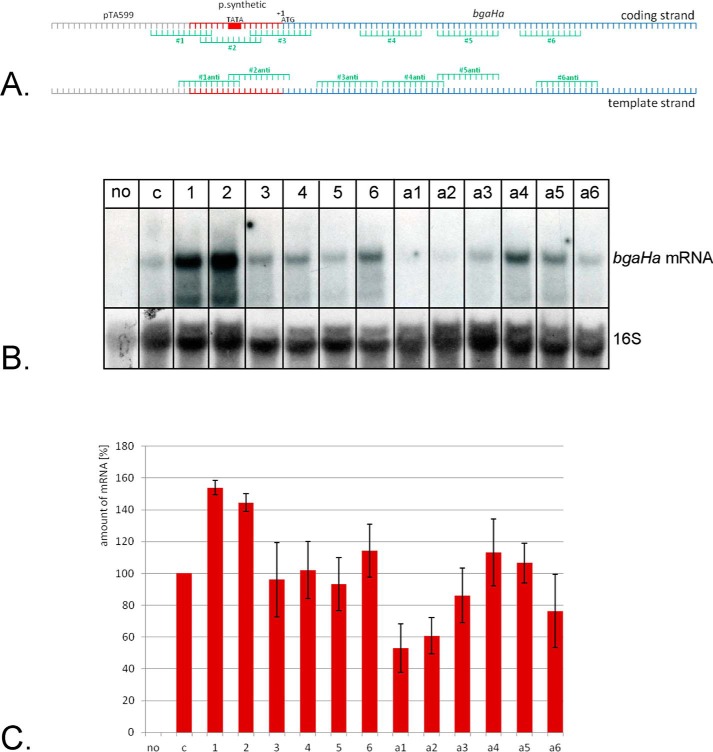
**Using CRISPRi to repress *bgaHa* gene expression.** *A,* location of crRNA binding regions in the *bgaHa* gene. Both strands of the *bgaHa* gene are shown. crRNAs were designed to target the coding strand (*1–6*) or the template strand (#*1anti*-#*6anti*), genes for the crRNAs were cloned into pTA232, yielding plasmids pTA232-telebga#1–6 and pTA232-telebga#1–6anti. The *Haloferax* CRISPR-Cas system recognizes six different PAMs allowing to direct crRNAs to the desired sequence. The *bgaHa* mRNA is transcribed as leaderless mRNA starting directly with the ATG of the frame (indicated by +1 ATG above the coding strand). The coding region is shown in *blue*. The location of the TATA box is indicated with “TATA” and a *red box. B,* Northern blot analysis of target gene expression. RNA was isolated from strains expressing the different crRNAs (*lanes 1–a6*), separated on 0.8% denaturing agarose gels and transferred to membranes. Hybridization with a probe against the *bgaHa* mRNA shows that crRNA #1anti (*lane a1*) and #2anti (*lane a2*) have a strong repression effect (*upper panel*). The membrane was also hybridized with a probe against the 16S rRNA to determine the amount of RNA loaded in each lane (*lower panel*). *Lane no,* RNA isolated from HV30 cells without pTA599 (no *bgaHa* gene); *lane c*, RNA isolated from HV30 × pTA599 cells expressing a crRNA not targeting the reporter gene; *lanes 1–a6,* RNA isolated from HV30 strains expressing crRNAs #1, #2, #3, #4, #5, #6, #1anti, #2anti, #3anti, #4anti, #5anti, and #6anti, respectively. *C,* repression efficiency of different crRNAs. The amount of *bgaHa* mRNA was measured and set in relationship to the amount of 16S rRNA in each RNA fraction, the mRNA amount in strain HV30 × pTA599 expressing a crRNA not targeting the reporter gene (*c*) was set to 100%. The amount of mRNA is indicated on the *y* axis in %, the crRNAs used are shown on the *x* axis. The strongest repression effects were achieved with crRNAs targeting the template strand (#1anti represses down to 53% and #2anti down to 61%). Shown is the amount of *bgaHa* mRNA in *c*: HV30 × pTA599 expressing a crRNA not targeting the reporter gene; *no*, strain HV30; *1-a6*, HV30 × pTA599 strains expressing crRNAs #1, #2, #3, #4, #5, #6, #1anti, #2anti, #3anti, #4anti, #5anti, and #6anti, respectively.

To increase the number of Cascade complexes containing the crRNA that targets the gene to be repressed we next wanted to exclude the endogenous crRNAs. To that end we deleted the *cas6b* gene in strain HV29 resulting in strain HV30 (Δ*cas3*Δ*bgaH*Δ*cas6b*). Cas6b is the endonuclease that processes the primary transcript from the CRISPR locus into crRNAs and without the *cas6b* gene the 51 endogenous crRNAs encoded in the three *Haloferax* CRISPR loci cannot be generated (32). Thus the only crRNAs left in the cell to be incorporated into Cascade are the plasmid-encoded crRNAs. The HV30 strain was subsequently transformed with pTA599 to yield HV30 × pTA599, which was subsequently transformed with the crRNA expressing plasmids. For crRNA production a Cas6b independent crRNA expression system already established for *H. volcanii* is used (34). In this system crRNAs are expressed from the plasmid, resulting in a transcript that contains the crRNA flanked by two tRNA-like elements, which are processed by the tRNA processing enzymes RNase P and tRNase Z (38), generating a mature crRNA with exactly the same 5′ and 3′ ends as the “natural” crRNA (34) (Fig. 3*A*). RNA was isolated from this strain to monitor repression of the *bgaHa* expression. The different crRNAs clearly show different effects on *bgaHa* expression (Fig. 4*B*). Generally crRNAs binding to the coding strand result in no or very little repression. However, crRNAs targeting the template strand show clear repression of *bgaHa* expression (Fig. 4, *B* and *C*). In addition, crRNAs targeting the promoter region are more effective than crRNAs targeting the reading frame. The highest effect on the *bgaHa* mRNA can be found with crRNA #1anti, which targets the TATA box with a reduction down to 53%. A similar reduction was shown with the archaeal mRNA targeting system III-B. Here the mRNA targeted was reduced to 42% (16). To investigate whether the presence of the internal crRNAs would influence the down-regulation effect we repeated the experiments in the strain that still expresses Cas6b and therefore can generate the 51 internal crRNAs. Here, no repression effect was detected (data not shown). Thus the observed repression effects were achieved by deletion of the *cas6b* gene, confirming the hypothesis that the presence of additional crRNAs in the cell results in less or no repression. Taken together we could show that in strain HV30 targeting of the template strand in the promoter region results in a clear reduction of gene transcription.

##### Knockdown of a Chromosomal Gene

**FIGURE 5.**
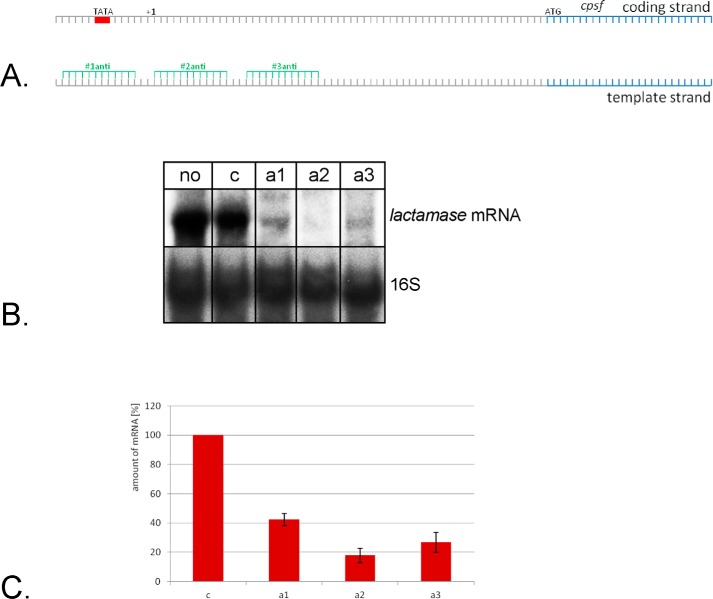
**Repression of the β-lactamase gene.** *A,* location of crRNA target sequences in the promoter region of the β-lactamase gene. Three crRNAs were designed to target the template strand. The TATA box is indicated as *red box*. The transcription start site is indicated by +*1 above* the coding strand and the start of the reading frame is indicated with ATG. The coding region is shown in *blue. B,* Northern blot analysis of β-lactamase gene repression. RNA was isolated, separated on 0.8% denaturing agarose gels, transferred to membranes, and hybridized with probes against the β-lactamase mRNA (*upper panel*). The membrane was also hybridized with a probe against the 16S rRNA to determine the amount of RNA loaded in each lane (*lower panel*). *Lane no*, RNA from strain HV30; *lane c*, RNA isolated from HV30 cells expressing a crRNA not targeting the β-lactamase gene; *lanes a1*, *a2*, and *a3*, RNA from HV30 strains expressing crRNAs #1anti, #2anti, and #3anti, respectively. *C*, repression efficiency of different crRNAs. The amount of the β-lactamase mRNA was measured and set into relationship to the amount of 16S rRNA in each RNA fraction, the RNA amount in strain HV30 expressing a crRNA not targeting the β-lactamase gene (*c*) was set to 100%. The amount of mRNA is indicated on the *y* axis in %, the crRNAs used are shown on the *x* axis. The strongest repression effect with 18% was achieved with crRNA #2anti targeting the template strand directly downstream of the transcription start site. Shown is the amount of β-lactamase mRNA in *c,* HV30 cells expressing a crRNA not targeting the β-lactamase gene; *a1–a3*, HV30 strains expressing crRNAs #1anti, #2anti, and #3anti, respectively.

Because down-regulation of a reporter gene on a plasmid worked well, we next wanted to repress a chromosomal gene. For this approach we chose as target the gene HVO_0874, which encodes a β-lactamase protein. Three crRNAs were designed and used to down-regulate expression of the target gene (Fig. 5*A*). Down-regulation of this chromosomal gene worked well (Fig. 5*B*) with repression effects down to 18% (Fig. 5*C*). Repression efficiency of the chromosomal gene is higher than that of the plasmid-encoded reporter gene. The plasmid used has a pHV1 origin of replication and thereby two copies per chromosome (39). This 2-fold number of targeting sequences might be the reason for the lower repression efficiency. Taken together a gene encoded on the chromosome can be efficiently down-regulated with the CRISPRi system.

##### Knockdown of a Cluster of Genes

**FIGURE 6.**
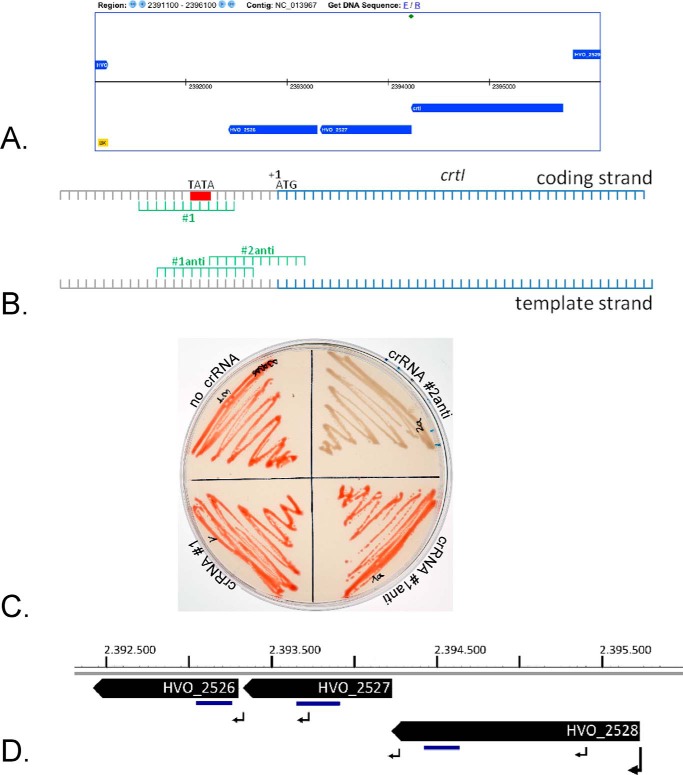
**Repression of carotenoid genes.** *A,* chromosomal location of the genes. The gene cluster encoding the three genes *crtI* (HVO_2528), HVO_2527, and HVO_2526 is shown (screenshot from the HaloLex server (47)). The first two genes (*crtI* and HVO_2527) overlap by four nucleotides. *B,* location of the crRNA target regions. Three crRNAs were designed to target the promoter region, two binding to the template strand (#*1anti* and #*2anti*) and one targeting the coding strand (#*1*). The TATA box is shown as a *red box*. The mRNA is leaderless starting directly with the ATG encoding the first amino acid (indicated by +1 ATG above the coding strand). The coding region is shown in *blue. C,* repression effect of the different crRNAs. The *red* color of the *Haloferax* cells is caused by carotenoids, therefore a reduction in carotenoid biosynthesis is easily monitored by a change of cell color. *Haloferax* cells that do not express a crRNA are *red* (*no crRNA*), as well as cells expressing crRNAs #1 and #1anti (*crRNA*#*1* and *crRNA* #*1anti*). Only cells expressing crRNA #2anti are *white* (*crRNA*#*2anti*). *D,* location of cDNAs generated with real time qRT-PCR and promoters. The locations of the main promoter and the weaker internal promoters are indicated by *arrows*. The cDNAs generated by real-time quantitative RT-PCR are shown as *blue bars below* the genes (see also Table 5). Only the minus strand of the genome is shown and the location of the genes on the chromosome is shown in nucleotides.

We next wanted to test down-regulation of a chromosomal gene cluster. Genes for synthesis of carotenoids are encoded by the gene cluster HVO_2526-HVO_2528 (Fig. 6*A*). Using RT-PCR we could show that all three genes are expressed as one multicistronic mRNA (data not shown). Next, three crRNAs targeting the promoter region of the first gene in this cluster were generated (Fig. 6*B*) and HV30 was transformed with these genes. Because carotenoids cause the red color of the *Haloferax* cells, targeting efficiency can be measured by monitoring the color of the cells. Normal expression of these genes results in the red color typical for *H. volcanii* cells (Fig. 6*C*), down-regulation would produce white cells. Cells expressing crRNAs #1 and #1anti are red, in contrast expression of crRNA #2anti results in white cells (Fig. 6*C*), showing that the latter was the most efficient crRNA for down-regulation. This experiment shows that a promoter for a cluster of genes can be down-regulated by targeting with crRNAs. To determine the amount of repression of all genes of the cluster we performed real-time quantitative RT-PCR. Compared with RNA from a strain that does not express a crRNA targeting the promoter of the *crtI* gene the mRNAs of all three cluster genes are clearly repressed in the CRISPRi strain (Table 2). The amount of repression is highest for the gene directly downstream of the promoter (*crtI* or HVO_2528) and slightly lower for HVO_2527 and HVO_2526. Transcription start site determination of *Haloferax* genes^3^ showed that weak internal promoters for the two downstream genes (HVO_2527 and HVO_2526) are present in the first two genes (Fig. 6*D*), suggesting that additional transcription from these promoters might be the cause for the slightly higher mRNA levels of the two downstream genes.

**TABLE 2:** **Targeting the promoter of a gene cluster results in repression of all genes present in the cluster** A crRNA targeting the promoter of the first gene in the carotenoid gene cluster reduces expression of all three genes in the cluster. The gene directly downstream of the targeted promoter (HVO_2528) shows the strongest repression (8%), the repression effect on the two downstream genes (HVO_2527 and HVO_2526) is slightly less strong (28 and 34%, respectively). The gene column indicates the gene for which the amount of relative transcript level was measured; column relative transcript level, the amount of relative transcript level was determined (see ”Experimental Procedures“). Note that the gene cluster is encoded on the minus strand, therefore the first gene in the cluster is HVO_2528 (*crtI*) followed by HVO_2527 and HVO_2526 (Fig. 6).

Gene	Relative transcript level (%)	S.D.
HVO_2528 (*crtI*)	8	5
HVO_2527	28	3
HVO_2526	34	13

Taken together this confirms the above observations that in this system crRNAs targeting the template strand in the promoter region have the highest effect. This is in contrast to the CRISPR-Cas9 CRISPRi tool used in eukarya and bacteria and the type I-E CRISPRi system in bacteria, where the targeting efficiency in the promoter region is independent of the nature of the strand (14, 19, 20). In these systems the nature of the strand only makes a difference upon targeting of the coding region, here targeting of the coding strand is more efficient than targeting of the template strand (14, 19, 20). There are several potential reasons for the observed differences (i) in contrast to the hitherto reported CRISPRi systems we used for the first time the type I-B system and (ii) employed it for the first time in archaea. Differences between transcriptional regulatory processes have been reported in archaea, bacteria, and eukarya. However, to finally determine the cause for the differences more data about the systems are required.

##### Knockdown of an Essential Gene

**FIGURE 7.**
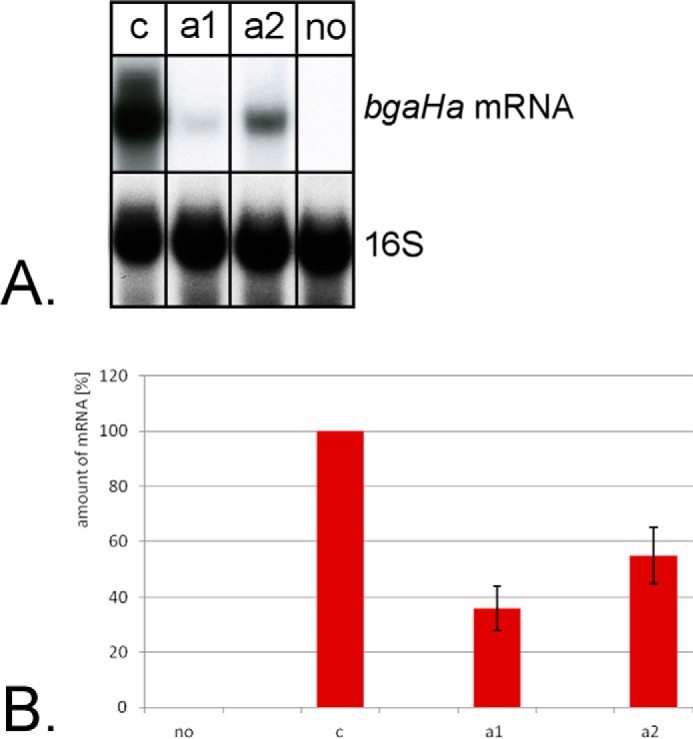
**crRNAs expressed from the S-plasmid.** *A,* Northern blot analysis of reporter gene expression. crRNA #1anti and #2anti that target the reporter gene β-galactosidase were expressed from the S-plasmid. RNA was isolated from HV33 cells expressing a control crRNA (*lane c*); from HV33 cells expressing crRNA #1anti and #2anti, respectively, against the reporter gene (*lane a1* and *a2*), from HV33 cells without *bgaHa* (*lane no*). After separation on a 0.8% denaturing agarose gel, the RNA was transferred to a membrane that was subsequently hybridized with a probe against the reporter gene mRNA (*upper panel*). The membrane was also hybridized with a probe against the 16S rRNA to determine the amount of RNA loaded in each lane (*lower panel*). *B,* effect of the S-plasmid expressed crRNAs on repression efficiencies. The amount of the reporter gene mRNA was measured and set into relationship to the amount of 16S rRNA in each RNA fraction, the RNA amount in strain HV33 expressing a crRNA not targeting the reporter gene (*c*) was set to 100%. The amount of mRNA is indicated on the *y* axis in %, the crRNAs used are shown on the *x* axis. Repression efficiencies without a crRNA and without bgaHa (*no*), with a crRNA that is not targeting the reporter gene (*c*), and with crRNAs targeting the gene (*a1* and *a2*) are shown. Repression of reporter gene transcription by #1anti reduces mRNA amounts down to 36%, whereas repression with crRNA #2anti is down to 55%.

**FIGURE 8.**
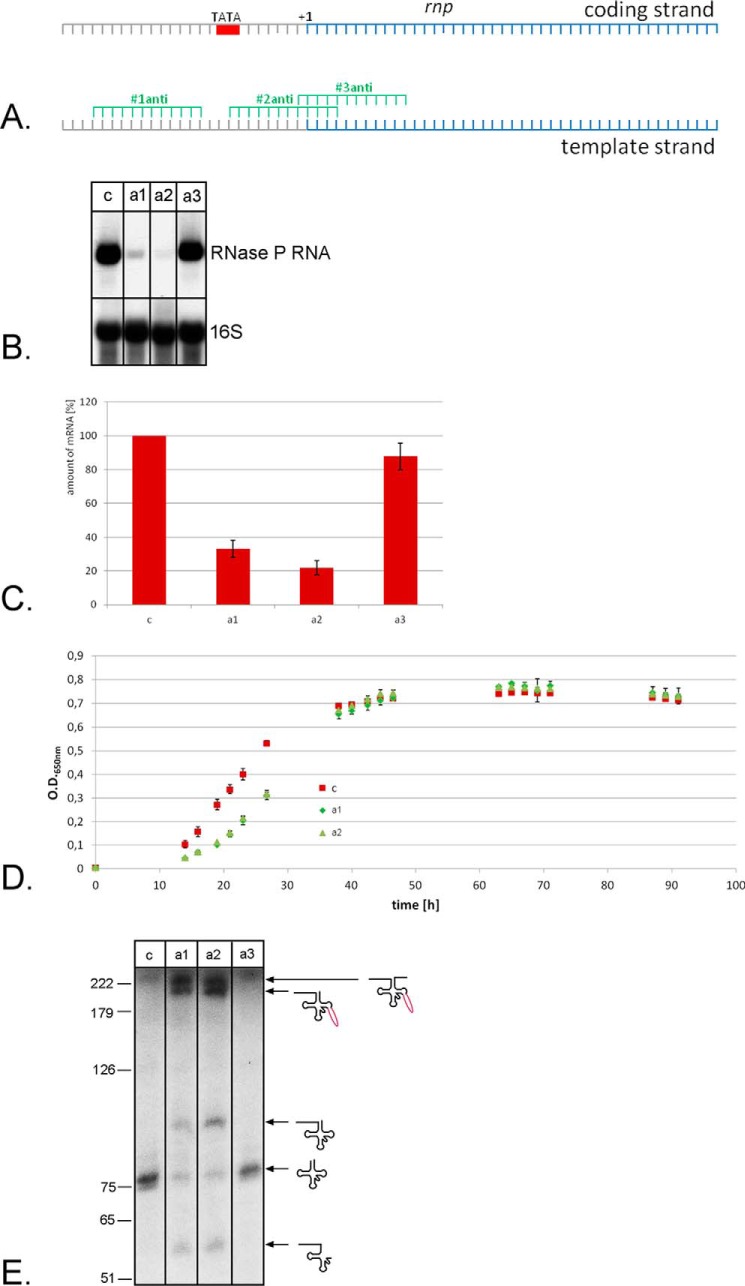
**Repression of the RNase P RNA gene.** *A,* location of crRNA target sequences in the promoter region of the RNase P RNA gene. Three crRNAs were designed targeting the template strand (#1anti, #2anti, and #3anti). S-plasmids expressing these crRNAs were generated and *Haloferax* strain HV33 × pTA927-cas6875 was transformed with these constructs. The TATA box is indicated as *red box*. The transcription start site is indicated by +1 *above* the coding strand. The coding region is shown in *blue. B*, Northern blot analysis of RNase P RNA gene repression. RNA was isolated, separated on 0.8% denaturing agarose gels, transferred to membranes, and hybridized with probes against the RNase P RNA (*upper panel*). The membrane was also hybridized with a probe against the 16S rRNA to determine the amount of RNA loaded in each lane (*lower panel*). *Lane c*, RNA isolated from HV33 cells expressing a crRNA not targeting the RNase P RNA gene; *lanes a1*, *a2,* and *a3*, RNA from HV33 strains expressing crRNAs #1anti, #2anti, and #3anti, respectively. *C,* repression efficiency of different crRNAs. The amount of RNase P RNA was measured and set into relationship to the amount of 16S rRNA in each RNA fraction, the RNA amount in strain HV33 expressing a crRNA not targeting the RNase P RNA gene (*c*) was set to 100%. The amount of mRNA is indicated on the *y* axis in %, the crRNAs used are shown on the *x* axis. The strongest repression effect was achieved with a crRNA targeting the template strand (#1anti with 22%). Shown is the amount of RNase P RNA in *c*: HV33 cells expressing a crRNA not targeting the RNase P RNA gene; *a1–a3*, HV33 strains expressing crRNAs #1anti, #2anti, and #3anti, respectively. *D,* repression of the RNase P RNA gene influences cell growth. Cells that express the crRNAs against the RNase P RNA gene promoter region (#1anti and #2anti) show a longer lag phase before the onset of growth. *E,* tRNA maturation is impeded in CRISPRi strains. Repression of the RNase P RNA gene results in accumulation of tRNA precursors and intermediates maintaining the 5′ leader. RNA was isolated from strains with and without crRNAs against the RNase P RNA gene, separated on an 8% PAGE, and transferred to a membrane. The membrane was hybridized with a probe against the 5′ leader and part of the 5′ end of the tRNA^Trp^, allowing to detect the 5′ leader and the mature tRNA. In cells expressing the crRNAs #1anti and #2anti maturation of tRNA^Trp^ is severely impaired. The full-length precursor containing the 5′ leader, intron, and 3′ trailer sequences is visible. The 3′ trailer can be removed from the precursor, because an intermediate RNA with 5′ leader, tRNA and intron is detected (193 nucleotides). Interestingly, the intron can be removed from the 5′ unprocessed precursor yielding a tRNA with 5′ leader (91 nucleotides). Only very little mature tRNA is visible. *Lane c*, RNA from a strain without a crRNA against the RNase P RNA gene, lanes: *a1*, *a2,* and *a3*, RNA from strains expressing the crRNAs #1anti, #2anti, and #3anti, respectively. A DNA size marker is shown at the *left* in nucleotides. Precursor and processing products are shown schematically at the *right*.

The tool for down-regulation of genes is especially interesting for the investigation of essential genes. Because we are interested in tRNA processing enzymes, we next aimed to repress the gene for the RNA subunit of the 5′ tRNA processing enzyme RNase P. For the previous experiments we used the crRNA expression system that generates a transcript containing the crRNA flanked by two tRNA-like elements, which are processed by the tRNA processing enzymes RNase P and tRNase Z (38) (Fig. 3*A*). For the analysis of a tRNA processing enzyme this system is not optimal, because the down-regulation of the tRNA 5′ processing enzyme RNase P would result in less crRNA production. Therefore we generated a minimal CRISPR locus, termed CRISPR locus S (for synthetic), encoded on a plasmid (termed S-plasmid). The S-plasmid contains the promoter and spacer sequences flanked by repeats from the *Haloferax* CRISPR locus C (Fig. 3*B*). The crRNAs are generated by Cas6b processing of the repeat sequences, for this approach we generated strain HV33, which has all internal CRISPR loci and all *cas* genes deleted. The *cas6b* gene and the *cas* genes required for CRISPRi (*cas5*, *cas7*, and *cas8b*) are expressed from a plasmid (HV33 × pTA927-cas6875). The S-plasmid was first used against the reporter gene to investigate its efficiency. The two crRNAs #1anti and #2anti (Fig. 4*A*), shown previously to be the most effective, were expressed from the S-plasmid and the effect on reporter gene expression was measured (Fig. 7). The repression effect of the S-plasmid-expressed crRNAs was slightly stronger than the effect with the previous expression system. crRNA #1anti had an efficiency with the previously used tRNA-like element system of 53%, with the new S-plasmid system efficiency was 36%. crRNA #2anti had an efficiency with the tRNA-like element system of 61% and with the S-plasmid system 55%. Next, three crRNAs targeting the promoter region of the RNase P RNA gene were designed (Fig. 8*A*), S-plasmids for their expression were generated and *Haloferax* cells were transformed with these constructs. Northern blot analyses showed that the crRNA targeting the template strand directly downstream of the TATA box worked best (Fig. 8*B*). The highest repression observed was a down-regulation to 22% of the wild type RNA level (Fig. 8*C*). Cells expressing the crRNAs have a longer lag phase (Fig. 8*D*) and are clearly impaired in tRNA processing (Fig. 8*E*). Taken together the CRISPRi system is an effective tool to down-regulate essential genes and thereby to be able to investigate essential genes *in vivo*.

##### Addition of a Cas3 Protein Variant

**FIGURE 9.**
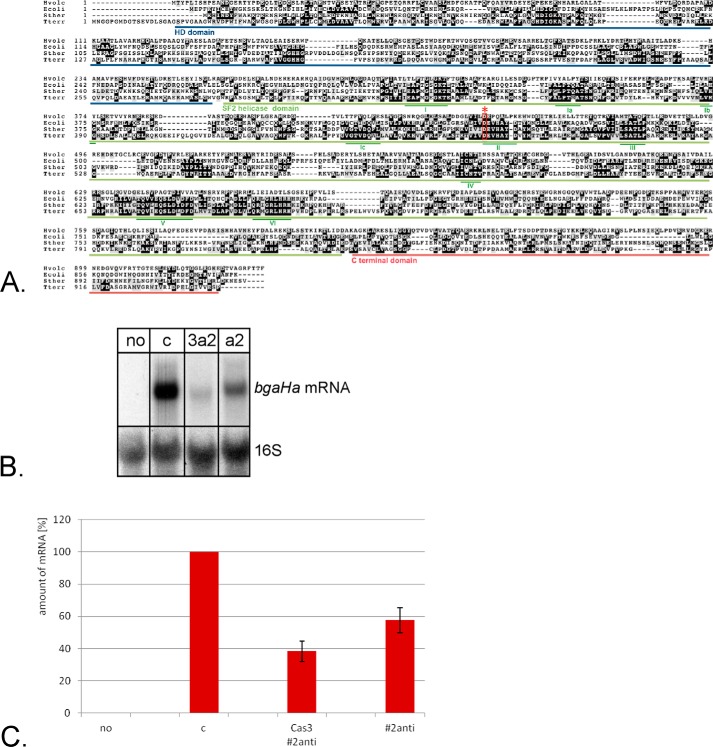
**Enhancing the CRISPRi effect with a Cas3 variant.** *A,* alignment of Cas3 proteins. The Cas3 protein sequences from *Thermobaculum terrenum* (*Tterr*), *S. thermophilus* (*Sther*), *E. coli,* and *H. volcanii* (*Hvolc*) were aligned. The different domains are underlined in different colors: *blue*, HD domain; *green*, SF2 helicase domain; and *red*, C-terminal domain (48). The motifs in the helicase domain are also shown (I-VI) (48). The Cas3 variant used here (D444A) has a mutation in motif II of the helicase domain, indicated by a *red asterisk. B,* effect of the Cas3 variant on repression efficiencies. *Haloferax* strain HV30 × pTA599 was transformed with pTA927-cas3D444A and subsequently with the plasmid expressing the crRNA against the *bgaHa* promoter region (#2anti). RNA was isolated from HV30 cells without pTA599 (no *bgaHa* gene) (*lane no*); from HV30 cells with pTA599 expressing the control crRNA (*lane c*), from HV30 cells with pTA599 expressing the #2anti crRNA (*lane a2*), and from HV30 × pTA599 cells expressing the Cas3 variant as well as the #2anti crRNA (*lane 3a2*). After separation of the RNA on a 0.8% denaturing agarose gel, the RNA was transferred to a membrane, which was subsequently hybridized with a probe against the *bgaHa* mRNA (*upper panel*). The membrane was also hybridized with a probe against the 16S rRNA to determine the amount of RNA loaded in each lane (*lower panel*). *C,* repression efficiencies. The amount of *bgaHa* mRNA was measured and set in relationship to the amount of 16S rRNA in each RNA fraction, the mRNA amount in strain HV30 × pTA599 expressing a crRNA not targeting the reporter gene (*c*) was set to 100%. The amount of mRNA is indicated on the *y* axis in %, the crRNAs used are shown on the *x* axis. Repression of the *bgaHa* gene with crRNA #2anti leads in this experiment to 58% mRNA (compared with Fig. 1, where #2anti lead to 61% repression, the deviation by 3% is in the range of the standard deviation). Repression with #2anti in the presence of the Cas3 variant further reduces mRNA amounts down to 38%. Thus, addition of the Cas3 variant results in a further reduction by 20%. Amount of *bgaHa* mRNA in *no lane*: strain HV30 without pTA599; *c,* HV30 × pTA599 cells expressing a crRNA not targeting the reporter gene; *Cas3*#*2anti*, HV30 × pTA599 × pTA927-cas3D444A expressing crRNA #2anti; *#2anti,* HV30 × pTA599 strains expressing crRNA #2anti.

To further enhance the knockdown effect we wanted to express a Cas3 protein variant that would bind to but not process the DNA target. If binding of Cas3 to Cascade stabilizes the Cascade-crRNA-target complex, the repression effect might be enhanced. Studies with the bacterial Cas3 protein from *E. coli* and *Streptococcus thermophilus* showed that mutations in the helicase domain resulted in reduced ATP binding/hydrolysis and loss of interference (21, 22). Therefore we generated a Cas3 variant that had a conserved aspartate in motif II of the helicase domain (DE*X*D/H helicase motif) mutated to alanine (D444A) (Fig. 9*A*). Interference tests showed that this mutation indeed resulted in a loss of function in the interference reaction (40). To investigate whether addition of this Cas3 variant would enhance the CRISPRi effect, strain HV30 × pTA599 was transformed with the crRNA expressing plasmid targeting the *bgaHa* promoter region (#2anti) and the plasmid expressing the Cas3 variant. The amount of the target gene expression was compared in strains with and without the Cas3 variant (Fig. 9, *B* and *C*). The addition of the Cas3 mutant enhances the knockdown effect suggesting that this Cas3 variant is indeed still able to bind to Cascade and the DNA but is not able to cleave. This observation suggests that binding of the Cas3 variant stabilizes the binding of the crRNA-Cascade complex to the DNA target thereby preventing binding by transcription factors and RNA polymerase. Taken together the repression effect of the CRISPRi system can be enhanced by 20% by expressing the Cas3 D444A variant.

##### Optimizing the crRNA

The central molecule for this down-regulation approach is the crRNA. For the *Haloferax* CRISPR-Cas type I-B crRNA requirements have already been investigated to some extent (31, 34). The crRNA has to base pair perfectly in the seed region (the first 10 nucleotides of the spacer part of the crRNA with only a mismatch at position six allowed). The 5′ handle of the crRNA is essential and cannot be removed, whereas the complete 3′ handle can be removed without reducing the interference efficiency (34). To facilitate easier design of the crRNA we investigated whether the spacer part of the crRNA can be shortened. We removed 5, 10, and 15 nucleotides from the 3′ end of the spacer and tested the activity of the resulting crRNAs using the interference assay (Table 3). The crRNA can be shortened by removing 5 nucleotides of the 36-nucleotide long spacer sequence. However, further reduction of the spacer sequence renders the crRNA inactive. Thus the shortest efficient crRNA is a 39-nucleotide long crRNA with an 8-nucleotide 5′ handle and a 31-nucleotide spacer. To facilitate easier cloning we next examined whether the crRNA can be expressed from a plasmid with a synthetic promoter with termination by a synthetic terminator. The crRNA was cloned directly downstream of the synthetic promoter p.syn and upstream of a synthetic terminator yielding plasmid pTA232-chuck-C1–22 (Fig. 3*C*) (34). In contrast to the tRNA-like construct and the S-plasmid used before, this setup does not ensure production of exact 5′ and 3′ ends, but might produce additional nucleotides at the crRNA 5′ and 3′ ends, if the RNA polymerase adds unencoded nucleotides at the 5′ and 3′ ends. To determine the activity of such a crRNA we employed the plasmid interference test (Table 4). The crRNA is not effective enough in the interference reaction, it reduces the transformation efficiency only by factor 0.3. A successful interference reaction reduces the number of transformants by at least factor 0.01. crRNAs expressed from the tRNA-like construct processed by RNase P and tRNase Z reduce the number of transformants at least by factor 0.01, thus these results suggests that correct 5′ and/or 3′ ends are important for crRNA function.

**TABLE 3:** **The minimal length of the spacer is 31 nucleotides** To facilitate easy crRNA design we investigated the requirements for the spacer length as part of the crRNA. crRNAs with spacers shortened from 36 to 31, 26, and 21 nucleotides were tested for their interference activity using the plasmid invader. *Haloferax* strain Δ*cas6* was first transformed with the different crRNA expressing plasmids (pTA232-crRNAspacerΔ5, pTA232-crRNAspacerΔ10, and pTA232-crRNAspacerΔ15) and subsequently with the invader plasmid pTA409-PAM3-C1 (31). Cells that have an active interference system degrade the invader plasmid and cannot grow on selective medium, resulting in drastically reduced transformation rates (reduced by at least factor 0.01) (30). Only the crRNA that is shortened by 5 nucleotides is still active in the interference reaction, the crRNAs with the shorter spacers are not active.

Plasmid expressing crRNA	Spacer length	Reduction in transformation rate by factor
pTA232-crRNAspacerΔ5	31	0.001
pTA232-crRNAspacerΔ10	26	1. 9 (no reduction, no interference)
pTA232-crRNAspacerΔ15	21	1.3 (no reduction, no interference)

**TABLE 4:** **Expression of crRNAs using a synthetic promoter and terminator** A plasmid invader assay was used to determine the interference activity of crRNAs expressed from a synthetic promoter with termination by a synthetic terminator. *Haloferax* strain Δ*cas6* was first transformed with the crRNA expressing plasmid (pTA232-chuck-C1–22) and subsequently with the invader plasmid pTA409-PAM3-C1 (31). The expressed crRNA contains a spacer that targets the plasmid invader. If the crRNA is expressed in a functional form it should be active in the interference reaction and should reduce the transformation efficiency by a factor of at least 0.01 (30). However, the crRNA only reduces transformation rates by factor 0.3, thus this crRNA is not as effective as crRNAs expressed with tRNA-like elements or crRNAs expressed from an S-plasmid.

crRNA expressed	Reduction in transformation rate by factor
Chuck-C1–22	0.3

##### A Tool for Down-regulation of Genes

The CRISPRi approach harnessing the endogenous CRISPR-Cas type I-B is the optimal tool for down-regulating genes in archaeal cells harboring type I-B systems. Employing the CRISPR-Cas9 system in extremophilic archaea is not straightforward because Cas9 proteins adapted to extreme conditions like high salt concentrations have to be selected. Whereas the use of the endogenous CRISPR-Cas system only requires deletion of two *cas* genes (the ones for Cas3 and Cas6b) as demonstrated here. In addition the endogenous type I-B system of *Haloferax* has the advantage to be active with six different PAM sequences: TTC, ACT, TAA, TAT, TAG, and CAC, making the target site selection easy. For regulation of genes in *Haloferax* the optimal tool is according to results presented here for strain HV33 × pTA927–6875 expressing the targeting crRNA from an S-plasmid. The optimal crRNA should target the template strand at the promoter region or the transcription start site. Two or three different crRNAs should be tested because 100% faithful prediction for the efficiency of a given crRNA is not possible. Previous analysis of the effectivity of different crRNAs on interference already showed that different crRNAs show different interference activity (31). Spacer length, G/C content, and the nature of the PAM sequences could not be used to predict the efficiency of the crRNA. A similar observation was made in this study with the different crRNAs used. The strongest repression effect with this CRISPRi system was a transcription repression down to 8%. This amount of repression is stronger than the amount of degradation of transcripts observed by the archaeal mRNA targeting system type III-B (16) and is in the range of reduction of gene expression generated in eukaryotes with the use of miRNAs. This approach can be used in all archaeal organisms containing a type I-B system and constitutes a simple and efficient method for transcriptional silencing.

## Experimental Procedures

### 

#### 

##### Strains and Culture Conditions

*H. volcanii* strains H119 (strains used are listed in Table 5), HV28 (Δ*cas3*), HV29 (Δ*cas3*Δ*bgaH*), HV30 (Δ*cas3*Δ*bgaH*Δ*cas6b*), HV31 (Δ*pyrE2*, Δ*leuB*, Δ*trpA*, ΔHVO_2,385,045–2,386,660::trpA, ΔHVO_pHV4: 204,834–218,566), HV32 (Δ*pyrE2*, Δ*leuB*, Δ*trpA*, ΔHVO_2,385,045–2,386,660, ΔHVO_pHV4: 204,834–218,566), and HV33 (Δ*pyrE2*, Δ*leuB*, Δ*trpA*, ΔHVO_2,385,045–2,386,660, ΔHVO_pHV4: 204,834–218,566, Δ*bgaH*) were grown aerobically at 45 °C in Hv-YPC medium (41). *H. volcanii* strains containing plasmids were grown in Hv-Ca or Hv-min medium with the appropriate supplements. *E. coli* strains DH5α (Invitrogen) and GM121 (36) were grown aerobically at 37 °C in 2YT medium (42).

**TABLE 5:** **Strains used in this study**

Strains	Genotype	Source/Ref.
DH5α	F− ϕ80*lac*ZΔM15 Δ(*lac*ZYA-*arg*F) U169 *rec*A1 *end*A1 *hsd*R17 (rk−, mk+) *gal*- *pho*A *sup*E44 λ- *thi*-1 *gyr*A96 *rel*A1	Invitrogen
GM121	F− *dam-3 dcm-6 ara-14 fhuA31 galK2 galT22 hdsR3 lacY1 leu-6 thi-1 thr-1 tsx-78*	36
H119	Δ*pyrE2,* ΔtrpA, Δ*leu*B	41
Δ*cas6*	Δ*pyrE2*, Δ*leuB*, Δ*trpA*, Δ*cas6*	32
HV28	Δ*pyrE2*, Δ*leuB*, Δ*trpA*, Δ*cas3*	S. Fischer, J. Richter, and E. Fischer, AG Marchfelder, unpublished data
HV29	Δ*pyrE2*, Δ*leuB*, Δ*trpA*, Δ*cas3,* Δ*bgaH*	This study
HV30	Δ*pyrE2*, Δ*leuB*, Δ*trpA*, Δ*cas3,* Δ*cas6,* Δ*bgaH*	This study
HV31	Δ*pyrE2*, Δ*leuB*, Δ*trpA*, ΔHVO_2,385,045–2,386,660::trpA, ΔHVO_pHV4: 204,834–218,566	44
HV32	Δ*pyrE2*, Δ*leuB*, Δ*trpA*, ΔHVO_2,385,045–2,386,660, ΔHVO_pHV4: 204,834–218,566	This study
HV33	Δ*pyrE2*, Δ*leuB*, Δ*trpA,* Δ*bgaH*, ΔHVO_2,385,045–2,386,660, ΔHVO_pHV4: 204,834–218,566	This study

##### Construction of Plasmids and Transformation of H. volcanii

Plasmids for expressing crRNAs against the different genes were generated as follows (plasmids are listed in Table 6).

**TABLE 6:** **Plasmids used in this study**

Plasmids	Relevant properties	Source/Ref.
pTA232	Shuttle vector with *leu*B marker and pHV2 replication origin	41
pTA409	Shuttle vector with *pyrE2* marker and pHV1 replication origin	49
pTA599	Shuttle vector with *trpA* marker and pHV1 replication origin, containing the *bgaHa* gene	37
pTA617	Shuttle vector with up and downstream regions of *bgaH* and no replication origin for *Haloferax*	41
pTA927	Shuttle vector with *pyrE2* marker and pHV2 replication origin	37
pTA927-P.syn	Shuttle vector with *pyrE2* marker, pHV2 replication origin and P.synthetic promoter	This study
pTA927-Flag	Shuttle vector with *pyrE2* marker, pHV2 replication origin and a 3× FLAG tag	32
pTA927-Cas3-Flag-N	*cas3* gene fused to an N-terminal FLAG tag	50
pblue-*cas3*	*cas3* gene	40
pblue-*cas3D444A*	Gene for Cas3 variant D444A	40
pTA927-cas3D444A	Mutated *cas3* gene (D444A)	40
pTA409-PAM3	Spacer P1.1. downstream of PAM3 (TTC)	30
pTA409-PAM3-C1	Spacer C1 downstream of PAM3 (TTC)	31
pMA-RQ-telecrRNA	*E. coli* plasmid containing the promoter, crRNA flanked by t-elements and terminator, expressing the crRNA against spacer C1	34
pMA-RQ-telecrRNA19	*E. coli* plasmid containing the promoter, crRNA without 3′ handle flanked by t-elements, and terminator, expressing the crRNA against spacer C1	34
pMA-RQ-crRNAspacerΔ5	*E. coli* plasmid containing the promoter, crRNA without 3′ handle flanked by t-elements and terminator, expressing the crRNA against spacer C1 shortened by 5 nucleotides	This study
pMA-RQ-crRNAspacerΔ10	*E. coli* plasmid containing the promoter, crRNA without 3′ handle flanked by t-elements and terminator, expressing the crRNA against spacer C1 shortened by 10 nucleotides	This study
pMA-RQ-crRNAspacerΔ15	*E. coli* plasmid containing the promoter, crRNA without 3′ handle flanked by t-elements and terminator, expressing the crRNA against spacer C1 shortened by 15 nucleotides	This study
pMA-RQ-telebga#X	*E. coli* plasmid containing the promoter, crRNA without 3′ handle flanked by t-elements and terminator, expressing a crRNA against the coding strand in the *bgaHa* gene (numbers 1–6)	This study
pMA-RQ-telebga#Xanti	*E. coli* plasmid containing the promoter, crRNA without 3′ handle flanked by t-elements and terminator, expressing a crRNA against the template strand in the *bgaH*a gene (#1anti- #6anti)	This study
pMK-RQ-CRISPRS-bga#1anti	*E. coli* plasmid containing the promoter, spacer sequence flanked by *Haloferax* repeats and terminator, expressing a crRNA against the template strand in the *bgaHa* promoter	This study
pMK-RQ-CRISPRS-bga#2anti	*E. coli* plasmid containing the promoter, spacer sequence flanked by *Haloferax* repeats and terminator, expressing a crRNA against the template strand in the *bgaHa* promoter	This study
pMK-RQ-anti1PRNA	*E. coli* plasmid containing the promoter, spacer sequence flanked by *Haloferax* repeats and terminator, expressing a crRNA against the RNase P RNA gene	This study
pMK-RQ-anti2PRNA	*E. coli* plasmid containing the promoter, spacer sequence flanked by *Haloferax* repeats and terminator, expressing a crRNA against the RNase P RNA gene	This study
pMK-RQ-anti3PRNA	*E. coli* plasmid containing the promoter, spacer sequence flanked by *Haloferax* repeats and terminator, expressing a crRNA against the RNase P RNA gene	This study
pMA-RQ-telecrtI#1	*E. coli* plasmid containing the promoter, crRNA without 3′ handle flanked by t-elements and terminator, expressing a crRNA against the *crtI* promoter	This study
pMA-RQ-telecrtI#1anti	*E. coli* plasmid containing the promoter, crRNA without 3′ handle flanked by t-elements and terminator, expressing a crRNA against the *crtI* promoter	This study
pMA-RQ-telecrtI#2anti	*E. coli* plasmid containing the promoter, crRNA without 3′ handle flanked by t-elements and terminator, expressing a crRNA against the *crtI* promoter	This study
pMA-RQ-telecpsf#1anti	*E. coli* plasmid containing the promoter, crRNA without 3′ handle flanked by t-elements and terminator, expressing a crRNA against the *cpsf* promoter	This study
pMA-RQ-telecpsf#2anti	*E. coli* plasmid containing the promoter, crRNA without 3′ handle flanked by t-elements and terminator, expressing a crRNA against the *cpsf* promoter	This study
pMA-RQ-telecpsf#3anti	*E. coli* plasmid containing the promoter, crRNA without 3′ handle flanked by t-elements and terminator, expressing a crRNA against the *cpsf* promoter	This study
pMA-RQ-P.syn-C1–22-T.syn	*E. coli* plasmid containing the promoter, crRNA and terminator, expressing a crRNA against C1 with a 22-nucleotide long 3′ handle	This study
pTA232-telecrRNA	Plasmid containing the promoter, crRNA flanked by t-elements and terminator, expressing the crRNA against spacer C1	1
pTA232-telecrRNA19	Plasmid containing the promoter, crRNA without 3′ handle flanked by t-elements and terminator, expressing the crRNA against spacer C1	34
pTA232-crRNAspacerΔ5	Plasmid containing the promoter, crRNA without 3′ handle flanked by t-elements and terminator, expressing the crRNA against spacer C1, spacer sequence shortened by 5 nucleotides	This study
pTA232-crRNAspacerΔ10	Plasmid containing the promoter, crRNA flanked by t-elements and terminator, expressing the crRNA against spacer C1, spacer sequence shortened by 10 nt	This study
pTA232-crRNAspacerΔ15	Plasmid containing the promoter, crRNA flanked by t-elements and terminator, expressing the crRNA against spacer C1, spacer sequence shortened by 15 nucleotides	This study
pTA232-telebga#X	Plasmid containing the promoter, crRNA flanked by t-elements and terminator, expressing a crRNA against the coding strand in the *bgaHa* gene (#1 to #6)	This study
pTA232-telebga#Xanti	Plasmid containing the promoter, crRNA flanked by t-elements and terminator, expressing a crRNA against the template strand in the *bgaH*a gene (#1anti to #6anti)	This study
pTA232-CRISPRS-bga#1anti	Plasmid containing the promoter, spacer sequence flanked by *Haloferax* repeats and terminator, expressing a crRNA against the template strand in the *bgaHa* promoter	This study
pTA232-CRISPRS-bga#2anti	Plasmid containing the promoter, spacer sequence flanked by *Haloferax* repeats and terminator, expressing a crRNA against the template strand in the *bgaHa* promoter	This study
pTA232-CRISPRS-anti1PRNA	Plasmid containing the promoter, spacer sequence flanked by *Haloferax* repeats and terminator, expressing a crRNA against the RNase P RNA gene	This study
pTA232-CRISPRS-anti2PRNA	Plasmid containing the promoter, spacer sequence flanked by *Haloferax* repeats and terminator, expressing a crRNA against the RNase P RNA gene	This study
pTA232-CRISPRS-anti3PRNA	Plasmid containing the promoter, spacer sequence flanked by *Haloferax* repeats and terminator, expressing a crRNA against the RNase P RNA gene	This study
pTA232-telecrtI#1	Plasmid containing the promoter, crRNA flanked by t-elements and terminator, expressing a crRNA against the *crtI* promoter	This study
pTA232-telecrtI#1anti	Plasmid containing the promoter, crRNA flanked by t-elements and terminator, expressing a crRNA against the *crtI* promoter	This study
pTA232-telecrtI#2anti	Plasmid containing the promoter, crRNA flanked by t-elements and terminator, expressing a crRNA against the *crtI* promoter	This study
pTA232-telecpsf#1anti	Plasmid containing the promoter, crRNA flanked by t-elements and terminator, expressing a crRNA against the *cpsf* promoter	This study
pTA232-telecpsf#2anti	Plasmid containing the promoter, crRNA flanked by t-elements and terminator, expressing a crRNA against the *cpsf* promoter	This study
pTA232-telecpsf#3anti	Plasmid containing the promoter, crRNA flanked by t-elements and terminator, expressing a crRNA against the *cpsf* promoter	This study
pTA232-chuck-C1–22	Plasmid containing the promoter, crRNA against spacer C1 with 22-nucleotide long 3′ handle and terminator	
pTA232-P.syn-rnP#1anti-T.syn	Plasmid containing the promoter, crRNA against RNase P RNA and terminator	This study
pTA232-telecrRNA19	Plasmid containing the promoter, crRNA with no 3′ handle flanked by t-elements and terminator, expressing the crRNA against spacer C1	34
pTA927-*cas6875*	Shuttle vector with *pyrE2*-marker, pHV2 replication origin, P.*tnaA* tryptophanase promoter, T.syn terminator, genes for *cas5–8*	This study
pTA927-cas3D444A	Shuttle vector with *pyrE2*-marker, pHV2 replication origin, P.*tnaA* tryptophanase promoter, T.syn terminator, gene for Cas3 variant with D444A mutation	This study
pTA131-cas3geneupdo	*cas3* flanked by about 500 bp up and downstream regions each	This study
pTA131-cas3updo	Up and downstream regions of *cas3*	This study
pTA131-cas6updo	Up and downstream regions of *cas6*	32

##### Plasmids for crRNAs with t-Elements

crRNAs against the reporter gene *bgaHa*, the promoter region of a β-lactamase gene (HVO_0874), and the *crtI* gene (HVO_2528) were generated by inverse PCR with pMA-RQ-telecrRNA19 (34) as template (primers for *bgaHa,* crBga#1-#6iPCRup/crBga#1-#6iPCRdo and crBga#1anti-#6antiiPCRup/crBga#1anti-#6antiiPCRdo; for the β-lactamase gene, cpsf#1antiiPCRup/cpsf#1antiiPCRdo, cpsf#2antiiPCRup/cpsf#2antiiPCRdo, and cpsf#3antiiPCRup/cpsf#3antiiPCRdo; and for *crtI,* crPhyDe#1iPCRup/crPhyDe #1iPCRdo, crPhyDe#1antiiPCRup/crPhyDe#1antiiPCRdo, and crPhyDe#2antiiPCRup/crPhyDe#2antiiPCRdo). Primers used for iPCR omit the spacer for telecrRNA19 and contain the new spacer sequence (primer sequences are available upon request). The resulting plasmids contain a synthetic *Haloferax* promoter,^4^ the crRNA without 3′ handle, flanked by t-elements and a synthetic *Haloferax* terminator.^4^ Plasmids were digested with KpnI and BamHI to isolate the DNA fragment containing the complete insert. The resulting fragment was cloned into the *Haloferax* shuttle vector pTA232 (41), resulting in plasmids pTA232-telebga#X, pTA232-telebga#Xanti, pTA232-telecpsf#1–3anti, and pTA232-telecrtI#1-#2anti.

##### Plasmids Expressing crRNAs from Synthetic CRISPR Loci

The plasmids containing a minimal synthetic CRISPR-locus were ordered from GeneArt® and obtained as plasmids pMK-RQ-CRISPRS-bga#1anti, pMK-RQ-CRISPRS-bga#2anti, pMK-RQ-CRISPRS-anti1PRNA, pMK-RQ-CRISPRS-anti2PRNA, and pMK-RQ-CRISPRS-anti3PRNA. The plasmids contain the promoter of CRISPR-locus C, one spacer flanked by *Haloferax* repeats and a synthetic *Haloferax* terminator. The complete inserts containing spacers against *bgaHa* were excised by digestion with BamHI and EcoRV and cloned into the *Haloferax* shuttle vector pTA232, resulting in plasmids pTA232-CRISPRS-bga#1anti and pTA232-CRISPRS-bga#2anti. The inserts with spacers against RNase P RNA were obtained by digestion with BamHI and KpnI and ligated with pTA232, generating plasmids pTA232-CRISPRS-anti1PRNA, pTA232-CRISPRS-anti2PRNA, and pTA232-CRISPRS-anti3PRNA.

##### Plasmids Expressing crRNAs with Shortened Spacers

The plasmid containing the C1–22 was ordered from GeneArt® and obtained as plasmid pMA-RQ-chuck-crRNAC1–22-Syn, it contains a synthetic *Haloferax* promoter, the crRNA with a 22-nucleotide long 3′ handle and a synthetic *Haloferax* terminator. The plasmid was digested with KpnI and BamHI to isolate the DNA fragment containing the complete insert. The resulting fragment was cloned into the *Haloferax* shuttle vector pTA232, resulting in plasmid pTA232-chuck-C1–22. For the generation of plasmid pTA232-telebga#1anti (spacer #1anti contains KpnI restriction site) a PCR with the respective pMA-plasmid as template was performed amplifying the complete insert (from promoter to terminator) using the primers p.syn-fw and t.syn-rev. The purified PCR product was ligated with pTA232 (EcoRV digested). The three crRNA mutants with shortened spacers (Δ5, Δ10, and Δ15) were generated by inverse PCR on pMA-telecrRNA19 using primer pairs itele1/del30, itele1/del31, and itele1/del32. Plasmids were digested with KpnI and BamHI to isolate the DNA fragment containing the complete insert. The resulting fragment was cloned into the *Haloferax* shuttle vector pTA232, resulting in plasmids pTA232-crRNAspacerΔ5-Δ15.

##### Cloning of cas3 Mutant Gene

The plasmid carrying the gene for the Cas3 variant D444A was generated as follows. The *cas3* gene was amplified by PCR from genomic DNA using oligonucleotides Cas3up-HindIII and Cas3do-FlagN. The resulting fragment was cloned into pBlueScriptII (Stratagene) linearized with EcoRV yielding pblue-*cas3*. This construct was used as template to introduce the mutation in amino acid 444 using the QuikChangeII Site-directed Mutagenesis Kit (Agilent Technologies). The resulting pblue-cas3D444A construct carrying the mutated *cas3* gene was digested with HindIII and SmaI and the *cas3* gene variant was subcloned into the plasmid pTA927-FLAG (32), yielding the construct pTA927-cas3D444A.

##### Transformation of H. volcanii

In preparation for transformation all plasmids were passaged through *E. coli* GM121 cells to avoid methylation. *Haloferax* cells were subsequently transformed using the polyethylene glycol method (41, 43).

##### Generation of Deletion Strains HV28-HV30, HV32–33

Deletion strains were generated using the pop-in/pop-out method as described previously; all strains are listed in Table 5 (35, 36).

##### Generation of Strain HV28

The *cas3* gene was PCR amplified with flanking regions from the chromosomal DNA of *H. volcanii* strain H119 using primer pairs Cas3KOup1-inf/Cas3KOup2-inf and Cas3KOdo1-inf/Cas3KOdo2-inf. The resulting PCR fragment was cloned into pTA131, yielding pTA131-cas3updo. H119 was subsequently transformed with this construct to allow integration (pop-in) of the plasmid into the genome. The subsequent selection for loss of the *pyrE2* marker by plating on 5-fluoroorotic acid (5-FOA) revealed pop-out mutants. Chromosomal DNA was isolated from the wild type and potential *cas3* deletion mutants. 10 μg of EcoRV-digested DNA was separated on a 0.8% agarose gel and transferred to a nylon membrane (Hybond^TM^-N, GE Healthcare). The *cas3* downstream region was amplified with primers Cas3KOup1-inf and Cas3Sondedo using the PCR DIG Probe Synthesis Kit (Roche) and used as a hybridization probe. Hybridization and detection were performed according to the DIG manual (DIG Luminescent Detection Kit, Roche Applied Science). The resulting strain was termed HV28.

##### Generation of Strain HV29

For the deletion of the *bgaH* gene, HV28 was transformed with the vector pTA617 (41), which carries the up- and downstream regions of *bgaH*, to allow integration (pop-in) into the genome. The subsequent selection for loss of the vector by plating on 5-FOA led to pop-out mutants. To confirm the removal of *bgaH*, Southern blot analysis was performed. Chromosomal DNA was isolated from the wild type and potential *bgaH* deletion mutants. 10 μg of SaII-digested DNA was separated on a 0.8% agarose gel and transferred to a nylon membrane (Hybond^TM^-N, GE Healthcare). A 400-bp fragment of the downstream region of *bgaH* was amplified using primers bgaHKODO-for and bgaHKODO-rev, the fragment was labeled using the PCR DIG Probe Synthesis Kit (Roche) and used as a hybridization probe. Hybridization and detection were performed according to the DIG manual (DIG Luminescent Detection Kit, Roche). The resulting strain was termed HV29.

##### Generation of Strain HV30

For deletion of the *cas6b* gene, HV29 was transformed with the plasmid pTA131-cas6updo (32) to achieve integration of the plasmid into the genome. Subsequent plating on 5-FOA generated pop-out clones that were further investigated by Southern blot analysis. 10 μg of SaII-digested genomic DNA was separated on a 0.8% agarose gel and transferred to a nylon membrane (Hybond^TM^-N, GE Healthcare). A 300-bp fragment of the upstream region of the *cas6* gene was amplified using primers Cas6Sonde-up and iPCRCas6KOup2. The fragment was radioactively labeled using [α-^32^P]dCTP and the DECAprime II DNA labeling kit (Life Technologies) and subsequently used as hybridization probe. The resulting strain was termed HV30.

##### Generation of Strain HV32

For the deletion of the *trpA* gene in HV31 (44), the strain was transformed with plasmid pTA131-Cupdo (34), which contains the up- and downstream regions of CRISPR-locus C for integration into the genome. Subsequent selection for loss of the *pyrE2* marker by plating on 5-FOA agar led to pop-out colonies. To confirm the removal of *trpA*, Southern blot analyses were performed. Genomic DNA from potential deletion mutants was digested with SacII, separated on a 0.8% agarose gel, and transferred to a nylon membrane (Hybond^TM^-N, GE Healthcare). A 250-bp fragment of the downstream region of locus C was amplified using primers Cdeldoi and DomitteC, radioactively labeled with [α-^32^P]dCTP and the DECAprime II DNA labeling kit (Life Technologies) and used as hybridization probe. The resulting strain was termed HV32.

##### Generation of Strain HV33

To delete the *bgaH* gene from HV32, the strain was transformed with the plasmid pTA617 (41) (which contains the up- and downstream regions of the *bgaH* gene) to achieve integration of the plasmid into the genome. Subsequent plating on 5-FOA agar generated pop-out colonies that were analyzed by Southern blot analysis. 10 μg of SalI-digested genomic DNA were separated on a 0.8% agarose gel and transferred to a nylon membrane (Hybond^TM^-N, GE Healthcare). Using primers bgaHKODO-for and bgaHKODO-rev, a 400-bp fragment of the downstream region of *bgaH* was amplified. The fragment was radioactively labeled with [α-^32^P]dCTP and the DECAprime II DNA labeling kit (Life Technologies) and used as a hybridization probe. The resulting strain was termed HV33.

##### Plasmid Invader Tests

For invader tests strain Δ*cas6* was transformed with the crRNA expressing plasmid and subsequently with the invader plasmid (pTA409-PAM3-C1 (31)). As a control reaction *Haloferax* cells expressing crRNAs were transformed with the vector without insert (pTA409). For interference tests with Δ*cas3* invader plasmid pTA409-PAM3 was used. Plasmids were passaged through *E. coli* GM121 cells (to avoid methylation) and then introduced into *Haloferax* cells using the PEG method (41, 43). To confirm a successful interference reaction, *H. volcanii* cells were transformed at least three times with the plasmid invader construct or the control vector. Transformations with at least a 100-fold reduction in transformation rates are considered successful interference reactions (30, 45).

##### Northern Blot Hybridization

Total RNA was isolated if not stated otherwise from exponentially growing *H. volcanii* cells as described (31). After separation of 10 μg of RNA (total RNA) on a 0.8% denaturing agarose gel or 8% PAGE, RNA molecules were transferred to nylon membranes (Hybond-N^+^ or Hybond-XL, GE Healthcare) and incubated with radioactively labeled DNA fragments. PCR fragments were labeled using a DECAprime II DNA labeling kit (Life Technologies). Oligonucleotides used as hybridization probes were radioactively labeled at the 5′ end with [γ-^32^P]ATP. To quantify the amount of RNA the membranes were exposed to imaging plates (BAS-MS, Fujifilm) and analyzed using the FLA-3000 scanner (software BASreader 3.14). The intensity of signals was measured with ImageJ. The signals of the *bgaHa*, β-*lactamase* transcripts, and RNase P RNA were put into relationship to the 16S rRNA signal, which was used as RNA loading control. To obtain the percentage of RNA in the strains expressing crRNAs, the amount of RNA (mRNA and RNase P RNA) in a strain expressing a non-targeting crRNA was set to 100% and the RNA amounts in the strains containing the crRNA were set in relationship to these data. All Northern blot analyses were performed at least three times.

##### RT-PCR and Real-time qRT-PCR Analyses

To verify the presence of a multicistronic mRNA comprising transcripts from the HVO_2528-HVO_2526 genes, RNA was isolated from exponentially growing *H. volcanii* cells as previously described (29). After digestion of 10 μg of total RNA with TURBO^TM^ DNase (ThermoFisher Scientific), cDNA synthesis was performed using primer 2526-RT-rev, which can anneal to the transcript of HVO_2526, and RevertAid Reverse Transcriptase (ThermoFisher Scientific), subsequently, PCR was carried out using primers 2528qRT-fw and 2526qRT-rev, primers bind to the sequence of HVO_2528 (forward) and HVO_2526 (reverse), respectively. RNA was isolated from *H. volcanii* strains expressing the crRNA #2anti targeting the promoter of the carotenoid gene cluster and from strains expressing a crRNA that does not target a gene in the carotenoid gene cluster (control). Cells were grown to exponential phase as previously described (29). After digestion of 10 μg of total RNA with TURBO^TM^ DNase (Thermo Fischer Scientific), cDNA was synthesized using random hexamer primers (ThermoFisher Scientific) and RevertAid Reverse Transcriptase (ThermoFisher Scientific). All real-time PCR were performed using the KAPA^TM^ SYBR® Fast Mastermix for Roche LightCycler (Kapa Biosystems) and the LightCycler® 480 System (Roche Applied Science). Primers used were 2528-qRT-fw-k and 2528-qRT-rev-k for HVO_2528, 2527-qRT-fw-k and 2527-qRT-ref-k for HVO_2527, 2526-qRT-fw-k and 2526-qRT-rev-k for HVO_2526, tsgA3-qRT-fw-k and tsgA3-rev-k for *tsgA3*, and trmB1-qRT-fw-k and trmB1-qRT-rev-k for *trmB1*. Cycling conditions were composed of 7 min at 95 °C, 20 cycles with denaturation at 95 °C for 30 s, primer annealing at 68 °C for 30 s, and elongation at 72 °C for 20 s. This was followed by 40 cycles at 95 °C for 30 s, 57 °C for 30 s, and 72 °C for 20 s, and a final elongation step at 72 °C for 5 min. Reactions were performed in triplicate in three independent experiments. The cDNAs for genes *tsgA3* and *trmB1* were used as references to normalize obtained data for HVO_2526–2528. Data obtained with RNA from the strain expressing the targeting crRNA were set in relationship to data from a strain expressing a non-targeting crRNA, the latter was set to 100%.

## Author Contributions

A. E. S. conducted the experiments and analyzed the results, A. M. conceived the idea for the project and coordinated the study, analyzed the results together with A. E. S., and wrote the paper.

## Footnotes

3 J. Babski, K. A. Haas, D. Näther-Schindler, F. Pfeiffer, K. U. Förstner, M. Hammelmann, R. Hilker, A. Becker, C. Sharma, A. Marchfelder, and J. Soppa, manuscript in revision.

4 A. Sabag-Daigle and C. J. Daniels, unpublished data.

## Footnotes

2 The abbreviations used are:

CRISPR-Cas: clustered regularly interspaced short palindromic repeats-CRISPR associated
PAM: protospacer adjacent motif
S-plasmid: synthetic CRISPR locus plasmid
5-FOA: 5-fluoroorotic acid
qRT: quantitative RT.
